# Evaluation of the need for the integration of a nurse specialist (EndoNurse) into the interdisciplinary care of patients with endometriosis: Cross-sectional study

**DOI:** 10.1016/j.ijnsa.2025.100425

**Published:** 2025-09-18

**Authors:** Stefan Lukac, Viktoria Maria Kässens, Anne Barzel, Tabea Kloss, Ina Mayer, Wolfgang Janni, Katharina Hancke, Davut Dayan

**Affiliations:** a Department of Obstetrics and Gynecology, University Hospital Ulm, Ulm, Germany; b Faculty of Medicine, University of Ulm, Ulm, Germany; c Department for General Practice and Primary Care, University Hospital Ulm, Ulm, Germany; d Nursing Science and Practice Development Unit, University Hospital Ulm, Ulm, Germany

**Keywords:** Endometriosis, Nursing expert, Education, Therapy, Support

## Abstract

**Background:**

Like other chronic diseases, endometriosis also requires long-term, individualized and multidisciplinary care. The integration of specialized nursing care has already been proven in other chronic diseases, but it is unclear whether and in which areas the integration of an endometriosis nurse specialist (EndoNurse) would be welcomed by patients with endometriosis.

**Objective:**

The aim of this study is to identify the need and acceptance of an integration of a nurse specialist into the care of patients with endometriosis and possible counselling topics.

**Design:**

Cross-sectional study in Germany.

**Setting and Participants:**

844 German-speaking patients with diagnosed endometriosis completed an online survey distributed via official platforms and social media.

**Methods:**

The current satisfaction and needs of patients with endometriosis were evaluated in five dimensions (Physical and psychological symptoms, therapy, lifestyle, sexuality, partnership, fertility, treatment wishes) similar to the Endometriosis Impact Questionnaire. In addition, questions regarding the need for optimalization along with acceptance and preferred areas of care by an EndoNurse were included. The evaluation was based on the 5-point Likert-scale.

**Results:**

844 participants were included in the final analysis. Only 138 (16.4 %) of them described their current care as good or very good. Fewer than one in five patients felt well or very well informed concerning the topics symptoms (200; 23.7 %), therapy (209; 24.8 %), sexuality and family planning (124; 14.7 %) as well as sport and nutrition (111; 13.1 %). 774 (91.7 %) of the participants would accept an integration of an EndoNurse in their treatment, particularly regarding the following topics: pain management (594; 70.4 %), nutrition (570; 67.5 %), sports and physiotherapy (567; 67.2 %), coping with everyday life (558; 65.8 %) and therapy options (547; 64.8 %). 474 (56.2 %) of the patients named physicians as their preferred source of information. Specially trained medical staff (EndoNurse) followed with 159 (18.8 %) votes, ahead of the internet (88; 10.4 %), self-help groups (41; 4.9 %), apps (37; 4.4 %) and others.

**Conclusions:**

Our study demonstrates a significant need for improvement in supportive endometriosis care. The involvement of a specialised nurse, EndoNurse, into care of patients with endometriosis is a concept that is useful and desirable for those affected in all evaluated areas.

**Registration:**

The study was approved by the Ethical Committee of the University of Ulm Nr. 364/23 and registered in the German Register of Clinical Studies under Nr. DRKS00033078.

**What is already known**

• Endometriosis is a chronic incurable condition requiring patient education and multidisciplinary care.
• The integration of nursing skills and advanced nurse practitioners in the management of other chronic diseases has positive impact.
• The satisfaction of patients with endometriosis regarding their current care and their opinion of endometriosis nurse specialists are not known.

Alt-text: Unlabelled box

**What this paper adds**

• The current standard of care for endometriosis does not sufficiently meet the needs of patients with endometriosis.
• Patients with endometriosis appreciate highly the integration of an EndoNurse as a core aspect of interdisciplinary care.
• According to the patients, EndoNurse support should focus on pain management, nutrition, sport and coping with everyday issues.

Alt-text: Unlabelled box

## 1 Background

Endometriosis is a benign but chronic disease that affects around 10 % of all women of reproductive age, but the grey numbers are higher (Bonavina and Taylor, 2022; Norton et al., 2020). The disease often leads not only to pain symptoms but also has a significant impact on social function and is reducing the patient’s quality of life (Della Corte et al., 2020; Sims et al., 2021; Ulrich et al., 2020; Van Barneveld et al., 2022). Furthermore, endometriosis places a burden on the healthcare system, comparable to other chronic diseases, such as Crohn's disease, diabetes or rheumatoid arthritis (Della Corte et al., 2020; Eisenberg et al., 2018; Nnoaham et al., 2011; Simoens et al., 2012).

The chronic nature of this disease requires long-term, specialized, individually adapted and multidisciplinary care (National Institute for Health and Care Excellence, 2017; Ulrich et al., 2020). Many parts of this long-term care require specialist nursing skills. The value of integrating nurse specialists into the care of patients with some other chronic diseases, such as breast cancer, chronic inflammatory bowel disease and diabetes, has already been proven and contributes significantly to higher patient satisfaction and an improved quality of life (Albert et al., 2011; Coulter et al., 2016; Mahony et al., 2019; Refeld, 2023; Smith et al., 2022; Stahlke et al., 2017). The tasks of a nurse specialist, for example Breast-care-Nurse, generally include the comprehensive care and support of patients from the initial assessment to information and advice on the disease itself and topics such as therapeutic options, pain management and supportive care (Hussain Rawther et al., 2020; Rosso et al., 2021; Stahlke et al., 2017; Tshiananga et al., 2012; White and Wilkes, 1999). They coordinate and optimize treatment procedures and carry out post-operative follow-up examinations. German nurse specialists are guided by international standards and do comparable work (Voigt et al., 2011). They act as a supportive link between patients and the specialist medical team and thus not only help to improve treatment outcomes but also reduce the burden on the clinic in terms of staff resources, time and length of stay (Coenen et al., 2017; Martinez-Vinson et al., 2020; Norton et al., 2020; Stahlke et al., 2017; Yu et al., 2023). Through targeted education and coordination by nurse specialists (Breast-Care-Nurse), the care and treatment compliance of breast cancer patients was also optimized in the long term (Albert et al., 2011; Hussain Rawther et al., 2020). Moreover, patients perceive their care as holistic and satisfactory with physical, psychological, emotional, social aspects (Smith et al., 2022; Stahlke et al., 2017).

The topic of holistic care and patient satisfaction along with its impact on improved quality of life in patients with endometriosis has not yet been sufficiently examined from a scientific perspective. Studies on the current gaps in care and the wishes of those affected are missing. Nursing care for patients with endometriosis is currently not defined or established in Germany in contrast to English-speaking countries, in which advanced nurse practitioners are involved in endometriosis care. Nevertheless, there are no clear definitions about the tasks of an EndoNurse in general or what patients expect from an EndoNurse at all. Therefore, our study focused on evaluating the needs of women with endometriosis as well as the acceptance of nursing care and areas where the skills of an EndoNurse could be most usefully applied.

## 2 Material and methods

### 2.1 Participants

This cross-sectional study was conducted between January 9th to March 10th, 2024. The study population comprises German-speaking, adult patients with clinically (magnetic resonance imaging, sonography) or histologically diagnosed endometriosis. Women younger than 18 years and those without diagnosed endometriosis or only isolated suspicion of endometriosis were excluded. The survey was conducted using the online platform LamaPoll, and all participants gave digital consent in line with the EU's General Data Protection Regulation (GDPR).

### 2.2 Data collection

Patients with endometriosis were contacted in various digital ways. Firstly, the Endometriosis Association of Germany, the official organization for patients with endometriosis, published the information and the link to our study on their official website for two weeks. In addition, 32 registered self-help groups for patients with endometriosis from all over Germany, listed on the website of the Endometriosis Association Germany e.V. with their contact data (Endometriose-Vereinigung Deutschland e.V., n.d.), were informed by email to the chair of their respective groups. They were asked to share the survey with the members of their group. Three chairs/self-help groups confirmed distribution of the study information. However, members of other self-help groups might have received the information about the study as well without any feedback being provided.

Further, we used social media to spread the study information. The EndoApp Team, provider of an officially approved digital health application (DiGA) for patients with confirmed endometriosis, and six “endometriosis influencers” were contacted on Instagram to recruit participants. Of those “endometriosis influencers” contacted on Instagram, only Ms. Vivian Vanessa Wagner, known on Instagram as “endoloewin”, with around 17 900 followers (January 2024), responded to the request and shared the study call once via her Instagram story. The EndoApp Team posted the information and the link to our survey twice via an Instagram story on their account, called “endo_app”, which has around 19 100 followers (January 2024). The information was online for 24 h in both cases. In addition, participation in eight online self-help groups on Facebook focusing on endometriosis was requested but only granted in four of these groups (“Endometriose ganzheitlich verstehen| umsetzen| leben”; “Endometriose – Gruppe für betroffene Frauen”; “Endometriose, Regelschmerzen, Frauenprobleme & Myome”; “Endometriose/ Adenomyose und ihre Begleiterkrankungen”). These groups have between 106 and 2618 members, whereby multiple membership in more than one group is possible. In each case, a one-off post was sent to the group with an invitation to the study and the link to the online survey.

### 2.3 Questionnaire and dimensions

As there is no questionnaire on the need for education and evaluation of nursing in endometriosis yet, we adhered to Moradi's Endometriosis Impact Questionnaire (EIQ) (Moradi et al., 2019) that measures a long term impact of endometriosis in the following areas: Impact of endometriosis on physical and psychosocial parameters, sexuality, intimate relationships, fertility, employment, financial and educational parameters and lifestyle. Based on the EIQ, we created an own questionnaire with the following parameters: general anthropometric data (age, height and weight to calculate the body mass index), social data (relationship status, highest level of education, nationality, religion and time since diagnosis) and partly modified dimensions of the EIQ (physical and psychological symptoms, therapy, lifestyle, sexuality, partnership, fertility, counselling wishes) (Appendix 1). In addition, questions were included on patient satisfaction with the current care and treatment as well as the level of education. The responses were scaled on the 5-point-Likert scale.

Finally, questions were added on current and desired sources of information regarding endometriosis, areas for improvement in education and nursing care, acceptance of support by an EndoNurse in the future (yes/I do not know/no) as well as areas where the EndoNurse could be integrated.

### 2.4 Statistical data analysis

The data collected was statistically analyzed after the survey ended. The cohort was characterized by descriptive statistics of the variables (frequency, median, range). The areas requiring clarification were described using the Likert scale and categorized according to priority.

### 2.5 Ethical approvement

The study was approved by the Ethical Committee of the University Ulm Nr. 364/23 and registered in the German Register of Clinical Studies under Nr. DRKS00033078.

## 3 Results

844 of 1036 participants were included in the final analysis, in line with the inclusion criteria. The median age was 31 years, and the median duration of disease was two years. The diagnosis was predominantly established during surgery. The majority of participants were in a relationship, had no children and reported being of normal weight, with an average body mass index of 23.3 kg/m2. The characteristics of the patient cohort are shown in Table 1.

**Table 1:** Characteristics of the patient´s with endometriosis in the survey:.

		844 (100 %)
**Age (years)**	Median (Range)	31 (18–58)
**Body Mass Index (kg/m^2^)**	Median (Range)	23.3 (15.4–48.3)
	missing	40 (4.7 %)
**Nationality**	German	790 (93.6 %)
	Other	54 (6.4 %)
**Highest level of education**	University degree	331 (39.2 %)
	apprenticeship	223 (26,4 %)
	A-levels	135 (16.0 %)
	Secondary school diploma	135 (16 %)
	Other	20 (2.4 %)
**Relationship status**	single	149 (17.7 %)
	in partnership	382 (45.3 %)
	married	271 (32.1 %)
	divorced	3 (0.3 %)
	missing	39 (4.6 %)
**Children**	yes	131 (15.5 %)
	No	674 (79.9 %)
	missing	39 (4.6 %)
**Number of children**	Median (Range)	1 (1–4)
**Time since diagnosis (years)**	Median (Range)	2 (0–28)
**Diagnosis established by**	Surgery	788 (93.3 %)
	Ultrasound	47 (5.6 %)
	Magnetic resonance tomography	9 (1.1 %)
**Art of the previous surgery**	therapeutic	617 (73.1 %)
	diagnostic	476 (56.4 %)
	no surgery	65 (7.7 %)
**Number of surgeries per patient**	Median (Range)	1 (1–11)
**Symptoms of endometriosis experienced**	dysmenorrhea	651 (77.1 %)
	dyspareunia	538 (63.7 %)
	dyschezia	424 (50.2 %)
	dysuria	262 (31 %)
	constipation	417 (49.4 %)
	flatulence	632 (74.9 %)
	back pain	632 (74.9)
	shoulder pain	220 (26.1)
	other	319 (37.8 %)
	none	16 (1.9 %)
**Elements in the current therapy**	Surgery	670 (73.1 %)
	Gestagen only pills	256 (30.3 %)
	Combined estrogen-gestagen pill	136 (16.1 %)
	hormonal intrauterine device	71 (8.4 %)
	hormone implant	10 (1.2 %)
	analgesics	554 (65,6 %)
	phytopharmaceuticals	122 (14,5 %)
	physiotherapy	166 (19,7 %)
	mediation, relaxation exercises	333 (39,5 %)
	physical measures (e.g. heat)	439 (52 %)
	dietary changes	353 (41,8 %)
	other	150 (17,8 %)

**Fig. 1:**
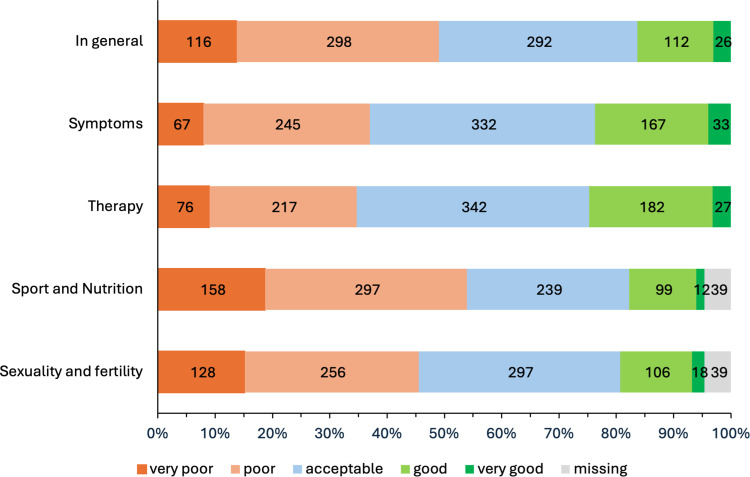
Satisfaction of patients with endometriosis with the evaluated topics in their current care.

Regarding satisfaction with current endometriosis care, only 138 (16.4 %) participants were satisfied, 414 (49 %) participants rated the received care as poor or very poor and 292 (36.4 %) participants as acceptable. Less than one in five patients felt well or very well informed concerning the topics symptoms (200; 23.7 %), therapy (209; 24.8 %), sexuality and family planning (124; 14.7 %) as well as sport and nutrition (111; 13.1 %) (Fig. 1).

About half of all patients (421; 49.9 %) reported following their doctor's treatment recommendations, 140 (16.6 %) only partially and 51 (6 %) tending to ignore them. 232 (27.5 %) women stated they were not taking any prescribed treatment. The 51 women mentioned gave the following reasons for non-compliance: 49 (96 %) named side effects, 25 (49 %) insufficient information from medical staff about the prescribed therapy and 7 (3.7 %) women mentioned high therapy costs.

**Fig. 2:**
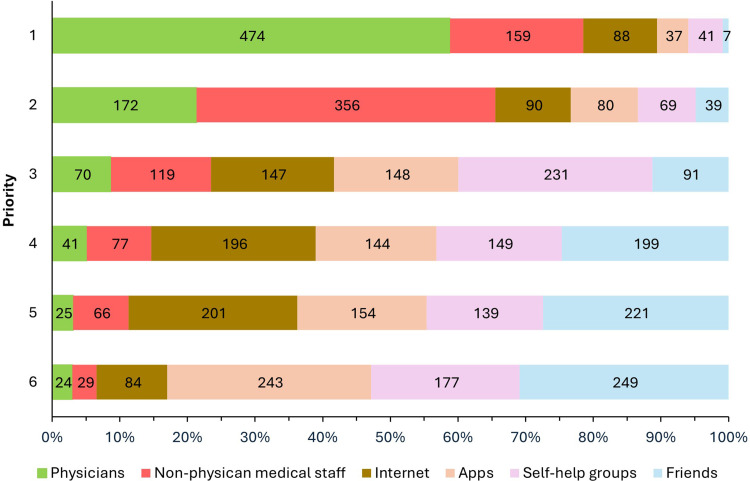
Desired sources of information for endometriosis treatment sorted by priority by patients with endometriosis.

Concerning getting information about endometriosis, participants used predominantly the internet (750; 88.9 %), followed by physicians (449; 53.2 %), apps (340; 40.3 %), self-help groups (318; 37.7 %), friends and acquaintances (232; 27.5 %) and other sources (141; 16.7 %). So far, nursing professionals have played a disproportionally small role in patient education (80; 9.5 %). If they had a choice, 474 (56.2 %) of the women asked would choose physicians as their preferred primary information source, whereas 159 (18.8 %) would favor a nurse specialist trained in endometriosis (EndoNurse), 88 (10.4 %) the internet, 41 (4.9 %) self-help groups, 37 (4.4 %) apps and 44 (5.3 %) patients would choose others. Asked to rank the different information sources, physicians were in first place, EndoNurses in second and friends and relatives in third (Fig. 2).

**Fig. 3:**
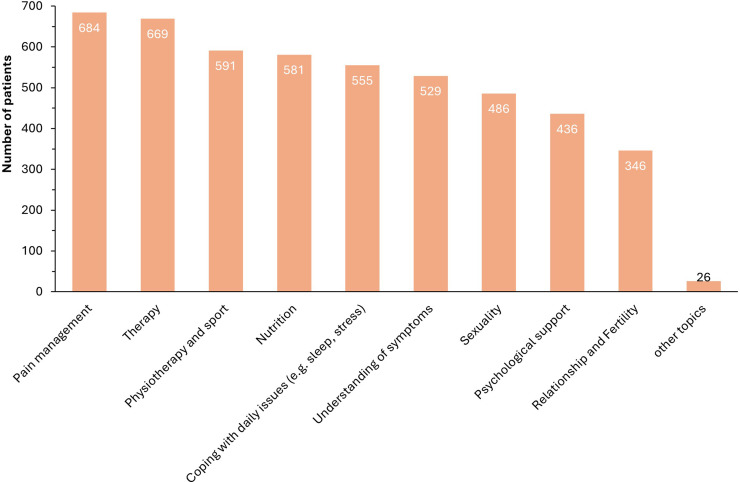
Desired areas for improvement in the endometriosis care.

**Fig. 4:**
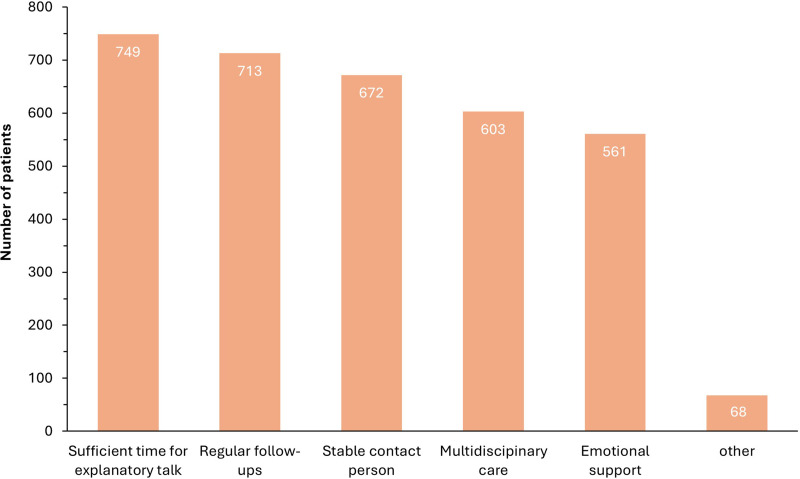
Desire elements in the care of patients with endometriosis.

Overall, participants' main concern was about pain and pain management (648; 81 %) alongside treatment options (669; 79.3 %). Other areas were also represented and are summarized in the Fig. 3. In responding to additional questions on how to improve their care, patients mentioned more time, regular check-ups and a stable contact person as the three most important aspects (Fig. 4).

**Fig. 5:**
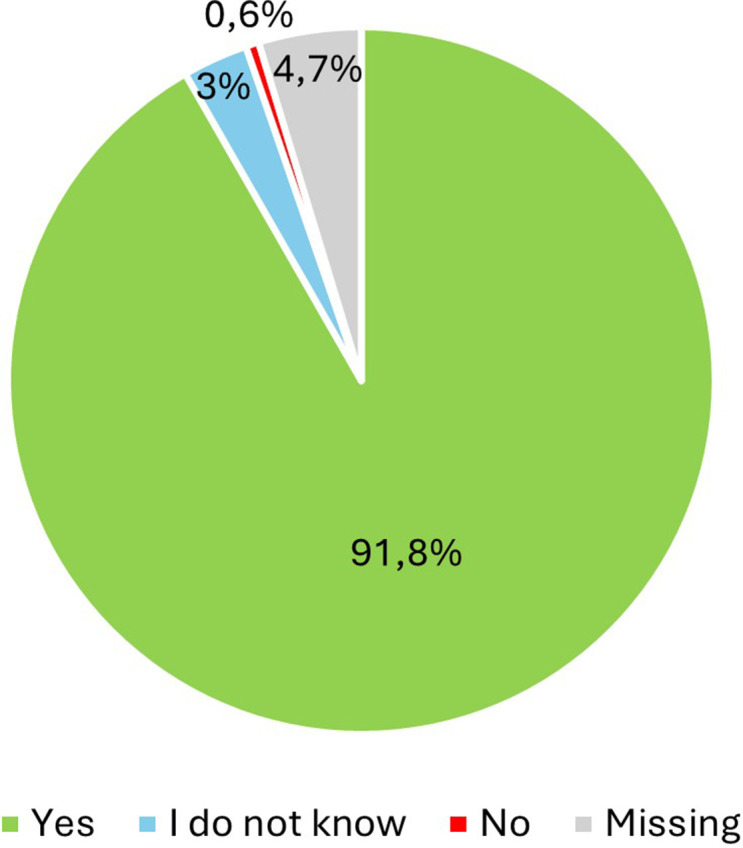
Acceptance of patients with endometriosis to be accompanied by an EndoNurse in their treatment.

**Fig. 6:**
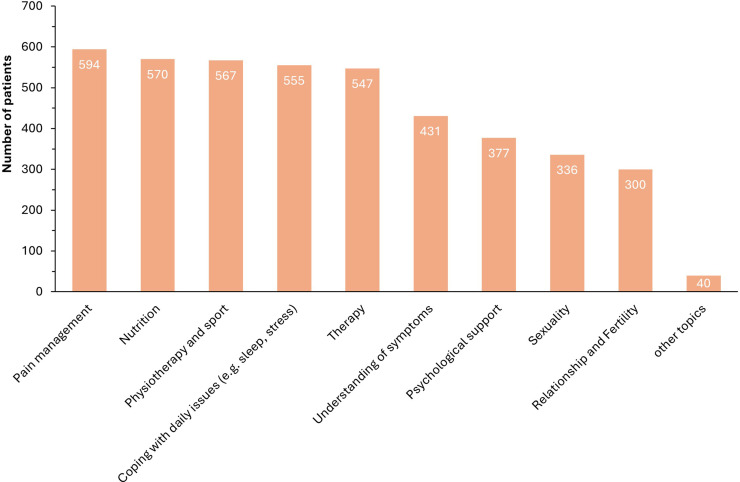
Areas of care in which patients with endometriosis would like to be supported by EndoNurse.

Finally, with regard to the EndoNurse, 774 (91.7 %) respondents could imagine being accompanied by an EndoNurse during their treatment and care in the future, 25 (3 %) did not know, 5 (0.6 %) would not be in favour of an EndoNurse and 40 (4.7 %) did not specify (Fig. 5). The preferred areas to be covered by an EndoNurse are shown in Fig. 6, with the main interest being in supportive measures: pain management (594; 70.4 %), nutrition (570; 67.5 %), sports and physiotherapy (567; 67.2 %), coping with everyday life (558; 65.8 %) and therapy options (547; 64.8 %).

## 4 Discussion

According to our knowledge, our study is world-wide the first one to evaluate the need for and preferred focus areas of a nurse specialist in endometriosis care, the EndoNurse, in a sizeable cohort of >800 women with endometriosis.

Our results show that there is an extensive need to improve care of patients with endometriosis, as satisfaction with the current management is low. Studies on women's perceptions of endometriosis care are scarce, as the majority of the studies focus solely on pain relief as an endpoint (Evans et al., 2022; Moradi et al., 2014). However, similar to our results, most women resported being generally dissatisfied with their care of endometriosis (Evans et al., 2022; Lukas et al., 2018).

Regarding the quality of care, respondents identified sufficient time for explanations, regular follow-ups, and a consistent point of contact as essential. Previous studies confirmed that it is not really the intensity of pain, but the duration of illness that was associated with dissatisfaction (Evans et al., 2022;Lukas et al., 2018). Besides endometriosis itself, there are further factors linked to decreased satisfaction, for example obstacles in accessing medical care, long waiting times, high treatment costs, stigmatization and inconsistent care (Culley et al., 2013; Evans et al., 2022). These results are consistent with the responses of our participants, highlighting the need for improvement. Especially in the area of extragenital endometriosis, ignorance of symptoms and a lack of understanding by physicians can negatively affect satisfaction of patients in terms of care provided (As-Sanie et al., 2019; Evans et al., 2022; Moradi et al., 2014; Lukac et al., 2024). In summary, continuous care in specialized units by professionally trained staff, combined with educating patients, is required to improve patient satisfaction.

In addition to the necessary medical expertise, empathy and trust are also crucial for successful treatment (Wattier, 2018) especially when addressing issues such as sexuality and psychological stress (Rossi et al., 2021; Ulrich, 2013). Our participants reported need for improvement in education concerning the topics sexuality, nutrition, sport, psychical well-being alongside understanding of symptoms and treatment. With reference to nursing care, building a relationship in a welcoming environment in which women can freely ask questions and express their own needs, concerns and expectations will lead to improved satisfaction and quality of life (Lukas et al., 2018).

Even in the digital age, women preferred a combination of physician and specially trained nursing staff as the primary source of information. Our results underline that, overall, patients are more inclined to seek special medical advice in this way and trust it more than an impersonal source of information such as the internet. Lukas et al. (2018) found that women who were satisfied with their treatment were more likely to describe communication with doctors as useful, while dissatisfied patients were more likely to use the internet as a source of information. This trend is also reflected in the results of this study. Better education can also lead to a more informed understanding of the disease and an improvement in quality of life (Ting et al., 2020). With regard to cancer, it is generally known that uncertainty about the disease is a significant cause of psychological stress, which has a negative impact on quality of life and promotes the use of avoidant coping strategies (Ting et al., 2020). This emphasizes the importance of patient education.

Many women, including the participants in this study, call for a holistic, caring and interdisciplinary approach to the treatment of endometriosis (Evans et al., 2022; Facchin et al., 2018). A multidisciplinary approach that includes physicians, responsible for medical and surgical treatment, nurse specialists and allied healthcare providers, such as psychologists and physiotherapists, is necessary to address the complex biopsychosocial needs of women affected by endometriosis (Agarwal et al., 2019; As-Sanie et al., 2019; Evans et al., 2022; Friedl et al., 2015; Lukac et al., 2023; Wattier, 2018). In addition, an interdisciplinary treatment concept provides many benefits such as pain relief, a reduction in visits to the emergency room, a reduction in the prevalence of concomitant diseases as well as an improvement in functional quality of life. This has already been proven for other chronic conditions (Allaire et al., 2018; As-Sanie et al., 2019). As part of a multidisciplinary team, the EndoNurse could play an important role and support patient-oriented education and thus contributing to greater patient satisfaction and compliance as well as better treatment outcomes, as it has already been demonstrated in other diseases (Gadot and Azuri, 2023; Gagan and Maybee, 2011; Mahony et al., 2019; Moore and McQuestion, 2012; Rosso et al., 2021; Tawfik et al., 2023).

The acceptance of an EndoNurse as part of an interdisciplinary treatment concept is very high among the participants in this survey. The fact that almost half of the patients in our study group would consult the endo-nurse in addition to the medical staff for primary information procurement and almost all of the women asked would accept additional consultations from an endo-nurse if these were offered, is remarkable, especially in view of the fact that the women have had no previous experience with an endo-nurse, as this concept is still in its infancy in Germany.

However, due to traditional role perceptions of healthcare professionals, it is essential to provide information about the individual role of an EndoNurse. The care provided by an EndoNurse is intended to be complementary, high-quality, specialist nursing care and the competences and independent skills of an EndoNurse should be recognized (Norton et al., 2020; Lukac et al., 2023). Therefore, in addition to the effective communication about the EndoNurse’s work as part of an interdisciplinary team, effective strategies should be developed for introducing the EndoNurse to patients, with the aim of promoting acceptance. Nevertheless, we expect comparable benefits and added values of the EndoNurse to those mentioned above of already existing nurse specialists such as Breast-Care-Nurses (Albert et al., 2011; Mahony et al., 2019; Stahlke et al., 2017).

Finally, the current healthcare system is challenging in terms of accessibility, staff shortages and financial pressure (Stahlke et al., 2017). However, as the study of Mahony et al. (2019) shows, the involvement of a nurse specialist can provide economic benefit by saving costs through reduced utilization of healthcare services, especially if patients get in touch with them at an early stage. This will be a beneficial add-on of implementing the EndoNurse.

## 5 Strengths and limitations

The size of our study population, and the fact that the analysis included only women with a confirmed diagnosis of endometriosis, are among the strengths of this study. Additionally, the multi-layered questionnaire included a wide range of topics regarding satisfaction with current care, the need for information, the acceptance of an EndoNurse and her areas of responsibility. Moreover, this study is one of very few addressing the important topic of patient care, satisfaction, gaps in care and wishes and needs of patients with endometriosis.

One limitation of our study is the unknown exact response rate as our sampling method limits the ability to calculate this rate. Furthermore, whether the EndoNurse will achieve the intended effect cannot be answered by our study, so further prospective studies are necessary for this.

## 6 Conclusions

Overall, there is a multifaceted lack of information in various care-related areas among patients with endometriosis. The integration of a nursing expert, EndoNurse, is a concept that is highly conceivable and desired by women with endometriosis. Together with the previously published literature, our results show that the implementation of an EndoNurse is sensible and desirable to address the diverse needs and wishes of women with endometriosis. We are convinced that the introduction of an EndoNurse will make a valuable contribution to the treatment and care of patients with endometriosis in a multidisciplinary team.

## Funding

This research did not receive any specific grant from funding agencies in the public, commercial or not-for-profit sectors.

### Data statement

The data that support the findings of this study are not openly available due to reasons of sensitivity and are available from the corresponding author upon reasonable request. Data are located in controlled access data storage at University Hospital Ulm.

## CRediT authorship contribution statement

**Stefan Lukac:** Writing – review & editing, Writing – original draft, Supervision, Resources, Project administration, Methodology, Investigation, Formal analysis, Data curation, Conceptualization. **Viktoria Maria Kässens:** Writing – review & editing, Writing – original draft, Project administration, Investigation, Formal analysis, Data curation. **Anne Barzel:** Writing – review & editing, Supervision, Methodology, Conceptualization. **Tabea Kloss:** Writing – review & editing, Methodology, Investigation. **Ina Mayer:** Writing – review & editing, Project administration, Investigation. **Wolfgang Janni:** Writing – review & editing, Supervision, Resources. **Katharina Hancke:** Writing – review & editing, Writing – original draft, Supervision, Methodology, Conceptualization. **Davut Dayan:** Writing – review & editing, Supervision, Resources, Project administration, Methodology, Investigation, Conceptualization.

## Declaration of competing interest

The authors declare that they have no known competing financial interests or personal relationships that could have appeared to influence the work reported in this paper.

## Appendix Supplementary materials

Image, application 1

Image, application 2
