# Supplementary material for: Evaluation of the need for the integration of a nurse specialist (EndoNurse) into the interdisciplinary care of patients with endometriosis: Cross-sectional study
Source: Int J Nurs Stud Adv. 2025 Sep 18;9:100425. doi: 10.1016/j.ijnsa.2025.100425 (PMC12538679; doi:10.1016/j.ijnsa.2025.100425)
Supplement: Supplementary file 1 [file mmc1.docx]

The data that support the findings of this study are not openly available due to reasons of sensitivity and are available from the corresponding author upon reasonable request. Data are located in controlled access data storage at University Hospital Ulm.
