# Supplementary material for: Evaluation of the need for the integration of a nurse specialist (EndoNurse) into the interdisciplinary care of patients with endometriosis: Cross-sectional study
Source: Int J Nurs Stud Adv. 2025 Sep 18;9:100425. doi: 10.1016/j.ijnsa.2025.100425 (PMC12538679; doi:10.1016/j.ijnsa.2025.100425)
Supplement: Supplementary file 2 [file mmc2.docx]

**Study questionnaire (translated from German original):**

1. How old are you?

Age (years)

2. What is your nationality? (e.g. German)

3. What is your highest level of education?

1. Secondary school certificate
2. A-levels
3. Training qualification
4. University degree
5. None of the given answer options

4. In which year were you diagnosed with endometriosis?

Year (e.g. 2023)

5. Have you already had surgery for your endometriosis?

1. Yes, as a diagnostic procedure (removal of tissue samples for diagnosis)
2. Yes, as a therapeutic procedure (for complete removal of the foci, possibly including

partial removal of bowel, bladder, diaphragm)

1. No

6. How many times have you been operated on for your endometriosis?

Number of surgical procedures

7. Which of the following confirmed your diagnosis of endometriosis?

1. Ultrasound
2. MRI
3. Palpation findings
4. So far there is only a suspicion of endometriosis

8. How do you rate your care in general since your diagnosis so far?

1. very good
2. good
3. acceptable
4. poor
5. very poor

9. Do you suffer from any of the following due to endometriosis? (multiple choice)

1. Pain during menstruation (dysmenorrhoea)
2. Pain when urinating (dysuria)
3. Pain during defecation (dyschezia)
4. Pain during sexual intercourse (dyspareunia)
5. Back pain
6. Shoulder pain
7. constipation
8. Flatulence
9. None
10. Other

10. On which side do you have the shoulder pain caused by endometriosis?

1. Left
2. both sides
3. Right

11. Other than those mentioned above, do you have any other physical symptoms of endometriosis?

12. Do you currently have one or more of the following mental health issues? (multiple choice)

1. Depression
2. Anxiety
3. Sleep disorders
4. Difficulty concentrating
5. Reduced energy
6. Difficulties in coping with everyday life
7. Eating disorder (anorexia, bulimia, binge eating)
8. Addiction (smoking, alcohol, drug, shopping, gambling, sports addiction, etc.)
9. Mood swings
10. Other
11. None

13. Other than those mentioned above, do you have any other mental health conditions or symptoms?

14. How well do you feel informed about how your physical and mental symptoms are related to endometriosis?

1. very good
2. good
3. acceptable
4. poor
5. very poor

15. Would you like more information about how endometriosis could be related to physical and mental health symptoms by healthcare professionals?

1. yes, definitely
2. yes, somewhat true
3. I do not know
4. no, not really true
5. no, does not apply at all

16. How is your endometriosis currently treated? (multiple choice)

1. Combined pill (with oestrogen)
2. Monopill (progestogen-only, without oestrogen)
3. Hormone coil
4. OP
5. Hormone injection (GnRH analogues)
6. Herbal preparation
7. painkillers
8. Physiotherapy
9. Physical measures (e.g. heat)
10. Relaxation exercises/meditation
11. Change of diet
12. Other

17. Are you taking a herbal supplement? If so, please provide its name,

name

18. Apart from the aspects mentioned above, how else do you manage your endometriosis?

19. How many hormonal treatments (various pills, hormone IUDs, GnRH analogues, etc.) have you used for your endometriosis?

number

20. How much of a financial burden is your treatment? (incl. medication, physiotherapy, travel costs, etc.)

1. very
2. somewhat
3. I don't know
4. hardly
5. not at all

21. How satisfied are you with your current treatment?

1. Very satisfied
2. Rather satisfied
3. Neither
4. Rather dissatisfied
5. Very dissatisfied

22. Do you follow the doctor's advice regarding your recommended management plan?

1. Yes
2. No
3. Partially
4. I have no prescribed therapy

23. What is preventing you from following the doctor's advice regarding your recommended treatment plan? (multiple choice)

1. Side effects
2. too costly
3. too complex
4. Insufficient medical information about the therapy
5. high costs
6. Other factors

24. Apart from the above, what is preventing you from following the doctor's advice regarding your recommended therapy?

25. How well informed do you feel about your treatment plan (surgical and medicinal etc.)?

1. very good
2. good
3. acceptable
4. poor
5. very poor

26. Would you like more information about your treatment plan from healthcare professionals?

1. yes, strongly agree
2. yes, somewhat true
3. I do not know
4. no, not really true
5. no, does not apply at all

27. How tall are you?

Height (in meters)

28. how much do you weigh?

Weight (in kilograms)

29. How often do you exercise?

1. Never
2. less than 1x per week
3. 1x per week
4. 2-3x per week
5. more than 3x per week

30. Do you smoke?

1. Yes
2. No

31. How often do you smoke?

1. Daily
2. Occasionally

32. How often do you drink alcohol?

1. Never
2. less than 1x per month
3. 1x per month
4. 2-3x per month
5. 1x per week
6. more often than 1x per week

33. Have you ever used cannabis?

1. Yes
2. No

34. How often do you use cannabis?

1. Less than once a month
2. 1x per month
3. 1x per week
4. more than 1x per week
5. daily
6. I have tried it before

35. In which form do you use cannabis?

1. Medically prescribed (therapeutic, on prescription)
2. on your own initiative (leisure)

36. Would you like addiction counselling or assistance with withdrawal symptoms?

1. Yes
2. No

37. How would you describe your dietary habits? (Multiple choice)

1. exclusively vegetarian
2. exclusively vegan
3. rich in vegetables
4. lots of meat (at least 3 times a week)
5. gluten-free
6. lactose-free
7. without further specification

38. Have you changed your diet after the diagnosis of endometriosis?

1. Yes
2. no

39. How often do you drink caffeinated drinks?

1. Never
2. Less than once a week
3. more often than 1x per week
4. 1x per day
5. several times a day

40. How would you classify your knowledge about sports and nutrition in relation to endometriosis care?

1. very good
2. good
3. acceptable
4. poor
5. very poor

41. Would you like more information on SPORT and NUTRITION in relation to endometriosis by healthcare professionals?

1. yes, strongly agree
2. yes, somewhat true
3. I do not know
4. no, not really true
5. no, does not apply at all

42. What is your religion/denomination?

1. Catholic
2. Protestant
3. Islam
4. None
5. Other

43. If other, please describe your religion.

44. What is your current relationship status?

1. Single
2. In a partnership
3. Married
4. Divorced
5. Widowed

45. Are you sexually active?

1. Yes
2. No

46. If yes, how frequently do you have sex?

1. daily
2. 2-4x per week
3. 1x per week
4. every 2 weeks
5. less often than every 2 weeks

47. How often do you avoid sexual intercourse?

1. Never
2. Rarely
3. Sometimes
4. Most of the time
5. Always

48. How satisfied are you with your sex life?

1. Very satisfied
2. Rather satisfied
3. Neither
4. Rather dissatisfied
5. Very dissatisfied

49. How often do you orgasm?

1. Never
2. Rarely
3. Sometimes
4. Most of the time
5. Always

50. What factors complicate your sex life? (multiple choice)

1. Pain
2. Fear of pain
3. Listlessness (reduced libido)
4. Exhaustion
5. Frustration
6. Self-doubt
7. Others
8. None

51. What other factors, apart from those mentioned above, complicate your sex life?

52. Which methods make your sex life easier? (multiple choice)

1. Lubricant
2. Painkillers
3. Change of position
4. Certain positions
5. Oral sex
6. Anal intercourse
7. Aids (e.g. Toys etc.)
8. Other

53. Which aids (e.g. toys etc.) make your sex life easier?

54. What other/additional methods not mentioned above make your sex life easier?

55. Does the topic of sexuality lead to conflicts in your partnership?

1. Yes
2. Sometimes
3. No

56. can you talk openly with your partner about your fears/worries regarding endometriosis?

1. Yes
2. No

57. Does he/she understand your symptoms?

1. Yes
2. No

58. Do you have children?

1. Yes
2. No

59. How many children do you have?

Number of children

60. Do you currently wish to have children?

1. Yes
2. No

61. Are you affiliated with a fertility centre?

1. Yes
2. No

62. Do you use psychological support services regarding your partnership and/or desire to have children?

1. Yes
2. No

63. What psychological support service do you use to discuss your relationship and/or desire to have children?

Name

64. How well do you feel informed about SEXUALITY and fertility with endometriosis?

1. Very good
2. Good
3. Acceptable
4. Poor
5. Very poor

65. Would you like more information about how your SEXUALITY and fertility can be impacted by endometriosis provided by healthcare professionals?

1. Yes, strongly agree
2. Yes, somewhat true
3. I do not know
4. No, rather not applicable
5. No, does not apply at all

66. Where do you get information from about endometriosis? (multiple choice)

1. (Gynaecologist) doctor
2. Non-physician medical staff (e.g. nurse, MFA etc.)
3. Self-help group
4. Friends/acquaintances
5. Internet
6. App
7. Other

67. Apart from the above, where did you find out about endometriosis?

68. Which source of information would you prefer to use, or which do you find best?

Rank the sources: top = best, bottom = worst

1. (Gynaecologist) doctor
2. Non-physician medical staff (e.g. nurse, MFA etc.)
3. Self-help group
4. Friends/acquaintances
5. Internet
6. App
7. Other

69. What would you wish for your care? (multiple choice)

1. Sufficient time for clarification
2. A stable contact person
3. Regular check-ups
4. Connection to other specialist disciplines
5. Emotional support
6. Other

70. Apart from the above, what would you like to see in your care?

71. In which areas would you like more counselling? (multiple choice)

1. Clinical picture and symptoms
2. Pain and pain management
3. Therapy (own therapy and possible therapy alternatives)
4. Sexuality
5. Desire to have children and family planning
6. Nutrition
7. Physiotherapy and sport
8. Coping with everyday life (stress reduction, sleep, etc.)
9. Psychological support services
10. Other services

72. In which other/additional areas would you like more counselling?

73. Would you make use of counselling sessions by specially trained medical staff in endometriosis (EndoNurse)?

1. Yes
2. no

74. In which of the areas mentioned would you make use of additional consultations with specially trained non-physicians/nursing specialist (EndoNurse)? (multiple choice)

1. Clinical picture and symptoms
2. Pain and pain management
3. Therapy (own therapy and possible therapy alternatives)
4. Sexuality
5. Desire to have children and family planning
6. Nutrition
7. Physiotherapy and sport
8. Coping with everyday life (stress reduction, sleep, etc.)
9. Psychological support services
10. Other services

75. In which other /additional areas than those mentioned above would you make use of consultations with specially trained non-physicians/nursing specialist (EndoNurse)?

76. Could you imagine receiving additional counselling in the future from medical nursing staff, specially trained in endometriosis to accompany and support you?

1. Yes, strongly agree
2. Yes, somewhat true
3. I do not know
4. No, rather not applicable
5. No, does not apply at all
